# Perioperative Cognitive Decline in Verbal Associative Memory Following Carotid Revascularization and Subsequent Risk of Adverse Events: Clinical Significance of the Standard Verbal Paired Associate Learning Test (S-PA)

**DOI:** 10.3390/brainsci16080877

**Published:** 2026-08-18

**Authors:** Yasunobu Nakai, Sakura Kurosu, Taisuke Akimoto, Go Ikeda, Tomoko Chujo, Midori Kusakabe, Takuma Hara, Kazuya Uemura, Tetsuya Yamamoto

**Affiliations:** 1 Department of Neurosurgery, Graduate School of Medicine, Yokohama City University, Yokohama 236-0004, Japan; akimoto.tai.ss@yokohama-cu.ac.jp (T.A.); y_neuros@yokohama-cu.ac.jp (T.Y.); 2 Department of Rehabilitation Therapy, Tokyo Metropolitan Rehabilitation Hospital, Tokyo 131-0034, Japan; st.sakura.6122@gmail.com; 3 Department of Neurosurgery, Dokkyo Medical University, Mibu 321-0293, Japan; g-ikeda@dokkyomed.ac.jp; 4 Department of Rehabilitation Therapy, Tsukuba Medical Center Hospital, Tsukuba 305-8558, Japan; chujo@tmch.or.jp (T.C.); kusakabe_midori@tmch.or.jp (M.K.); 5 Department of Neurosurgery, Tsukuba Medical Center Hospital, Tsukuba 305-8558, Japan; t9mhara@ybb.ne.jp (T.H.); kazuyaue@mac.com (K.U.)

**Keywords:** carotid artery stenosis, carotid endoarterectomy, carotid artery stenting, cerebrovascular vulnerability, cognitive impairment, standard verbal associate learning test (S-PA), stroke recurrence

## Abstract

**Highlights:**

**What are the main findings?**

- Perioperative cognitive changes following carotid revascularization (CEA or CAS) were prospectively evaluated using a comprehensive neuropsychological test battery, including the Standard Verbal Paired Associate Learning Test (S-PA).
- A decline in S-PA performance was associated with an increased risk of recurrent stroke, restenosis, and all-cause mortality during follow-up.

**What are the implications of the main findings?**

- Perioperative impairment in verbal associative memory may serve as a potential prognostic marker of cerebral vulnerability after carotid revascularization.
- Simple perioperative assessment using the S-PA may facilitate risk stratification and help identify patients who could benefit from more intensive secondary prevention strategies.

**Abstract:**

Background/Objectives: This study aimed to explore whether perioperative changes in cognitive function following carotid artery revascularization, including carotid endarterectomy (CEA) and carotid artery stenting (CAS), are associated with subsequent clinical outcomes, focusing specifically on the Standard Verbal Paired Associate Learning Test (S-PA). Methods: This prospective, single-center study enrolled 69 patients who underwent CEA or CAS between June 2018 and June 2024. Neuropsychological assessments (Raven’s Colored Progressive Matrices (RCPM), Kohs Block Design Test for IQ (Kohs-IQ), the Trail Making Test (TMT)-A/B, and the Standard Verbal Paired Associate Learning Test (S-PA)) were conducted preoperatively and within seven days postoperatively. Patient characteristics and diagnostic imaging results, including magnetic resonance imaging (MRI), magnetic resonance angiography (MRA), and single-photon emission computed tomography (SPECT), were evaluated. Multivariate Cox proportional hazards models and Kaplan–Meier survival analyses were used to identify predictors of adverse outcomes, defined as recurrent stroke, restenosis, or mortality, during outpatient follow-up. Results: Fifteen patients experienced adverse outcomes during follow-up. Perioperative neuropsychological assessment showed that 36 patients experienced S-PA decline, indicating a tendency towards lower mean cerebral blood flow. Receiver operating characteristic (ROC) curve for perioperative ΔS-PA decline associated with adverse outcomes. The area under the curve (AUC) was 0.728. The optimal cutoff determined by the Youden index was a decline of 2 points (ΔS-PA ≤ −2), yielding a sensitivity of 73.3% and specificity of 68.5%. In a time-to-event analysis, S-PA decline was associated with adverse outcomes in an unadjusted analysis. A similar trend was observed in limited multivariable models; however, effect estimates were imprecise (HR = 4.01, 95% CI: 1.01–15.91, *p* = 0.048). Conclusions: This study indicates that perioperative decline in S-PA may reflect underlying cerebrovascular vulnerability and may be associated with subsequent adverse outcomes. However, given the limited number of events and potential for residual confounding, these findings should be interpreted with caution. Larger multicenter studies are required to validate these findings.

## 1. Introduction

Carotid artery revascularization for carotid artery stenosis is considered an effective measure for preventing cerebral infarction, and carotid endarterectomy (CEA) or carotid artery stenting (CAS) are commonly performed [1]. Improvements in severe stenosis may improve cerebral blood flow and change cognitive function; however, some reports have revealed cognitive improvements after treatment [2,3,4,5,6,7,8]. We previously reported that the Standard Verbal Paired Associate Learning Test (S-PA) scores significantly worsened after carotid artery revascularization for symptomatic carotid stenosis [9]. This finding may indicate that S-PA is sensitive to subtle perioperative cognitive changes that are not readily captured by conventional imaging measures or broader neuropsychological assessments.

Previous studies have reported that post-stroke cognitive impairment (PSCI) affects patient quality of life and is a significant predictor of recurrent stroke and mortality [10,11,12]. Based on these observations, we hypothesized that perioperative cognitive decline after carotid artery revascularization is associated with long-term clinical outcomes. The primary aim of this exploratory study was to investigate whether a perioperative decline in neuropsychological test performance, particularly S-PA, is associated with long-term clinical outcomes.

## 2. Materials and Methods

### 2.1. Patient Selection

This prospective study enrolled patients who underwent CEA or CAS for carotid artery stenosis between June 2018 and June 2024 at our hospital. Patients were excluded from the present study if they were unable to undergo neuropsychological examination due to impaired physical conditions or consciousness disturbance, aphasia, or severe dementia, could not undergo the assessment due to perioperative management, or did not provide their informed consent. Patient characteristics including age, sex, medical history, smoking history, and diagnostic imaging information were collected. This study was approved by the institutional ethics committee (No. 2018-069).

### 2.2. Selection of Treatment Modality

The indications for the treatment of carotid artery stenosis at our hospital are based on the guidelines for the prevention of stroke in patients who have suffered a stroke or transient ischemic attack [13]. The treatment modality was selected based on the characteristics of the plaque on the stenotic part of the carotid artery; specifically, patients with unstable plaques were treated with CEA, while those with stable plaques were treated with CAS. The risks associated with each treatment modality were also considered [14].

### 2.3. Diagnostic Imaging

All patients underwent brain magnetic resonance imaging (MRI), magnetic resonance angiography (MRA), and single-photon emission computed tomography (SPECT) to assess cerebral blood flow both before and after treatment. Internal carotid artery stenosis was measured using 3D-CT angiography or cerebral angiography using the NASCET method [15]. Preoperative imaging was conducted approximately one-two weeks prior to surgery. Postoperative SPECT was conducted to ascend the hyperperfusion on the day of surgery, and brain MRI performed the following day to assess any ischemic complications revealed high-intensity areas (HIAs) on diffusion-weighted images (DWIs). Board-certified radiologists and neurosurgeons evaluated all the images.

### 2.4. Neuropsychological Examinations

In all cases, neuropsychological examinations were conducted approximately one to two weeks prior to surgery. Postoperative evaluation was started for CEA cases on the second day after surgery owing to the perioperative management of general anesthesia, whereas CAS cases were evaluated the day after surgery. Both were completed within seven days. In all cases, examinations were conducted by speech and occupational therapists. A battery of examinations were selected considering the physical burden during the perioperative period as follows: Raven’s Colored Progressive Matrices (RCPM), Kohs Block Design Test (KBDT), Trail Making Test—A and B (TMT-A and B), and Standard Verbal Paired Associate Learning Test (S-PA).

The RCPM was applied to assess the patients’ ability to solve problems and think logically using images and patterns without words. It does not depend on language or cultural knowledge. The test comprised several different pictures arranged in a grid or matrix, and the person taking the test had to find the missing piece from a list of options. The test took approximately 10–15 min [16].

The Kohs Block Design Test (KBDT) was used to assess fluid intelligence and visuospatial construction. Using 4–16 colored cubes, the patterns displayed on a series of test cards were replicated. The KBDT, adapted into sections of IQ tests (Kohs-IQ), takes approximately 20–50 min. In this study, Kohs-IQ score was used to represent the KBDT results [17].

The TMT-A and TMT-B were used to assess patients’ visual attention and task-switching abilities. TMT-A is instructed to connect a set of 25 numbers, while TMT-B is instructed to alternatively connect a set of 13 numbers and 12 kana letters as quickly as possible while maintaining accuracy. Scores were based on the time required to complete the test, with lower scores indicating better performance. This test takes approximately 5–10 min [18].

The S-PA was used to assess the patients’ verbal memory through the extensiveness of their vocabulary, their ability to find similarities among words, and their speed of handling and processing visual images. It was scored based on the number of related or unrelated words recalled from 10 pairs of auditory presentations [19]. The S-PA (related) items are easier than S-PA (unrelated) items, which evoked unrelated words. This test took approximately 10 min. In the present study, three test scores from the S-PA (related + unrelated) were used for analysis. Perioperative changes in S-PA performance were evaluated by comparison with preoperative and postoperative scores. In this exploratory study, S-PA decline was defined as any decrease in the S-PA score following surgery, and ΔS-PA was defined as the difference in perioperative S-PA scores.

### 2.5. Outcome Measures in Follow-Up

In the present study, adverse outcomes were defined as a composite of recurrent stroke (ischemic or hemorrhagic), restenosis requiring additional treatment, and all-cause mortality during follow-up. Stroke events were confirmed by clinical assessments and neuroimaging. Although restenosis is commonly detected as asymptomatic during routine follow-up, it has been included in adverse outcomes as a condition associated with a high risk of recurrent stroke. All-cause mortality included both vascular and non-vascular events.

### 2.6. Statistical Analysis

Pearson’s χ^2^ (Fisher’s exact test) and Wilcoxon/Kruskal–Wallis tests were conducted for comparisons between groups. Given the exploratory nature of this study and the limited number of outcome events, we employed a parsimonious approach to multivariate modeling. To minimize the risk of overfitting, variables included in the Cox proportional hazards models were used based on clinical relevance and prior literature, rather than statistical significance in the univariate analysis. The final multivariable Cox model included four covariates (age, symptomatic status, postoperative mCBF, and S-PA decline). The number of covariates was restricted to approximately one for 5–10 events, due to the limitations imposed by the sample size. Kaplan–Meier survival curves were constructed for adverse event-free survival, and differences between the groups were assessed using the log-rank test. All statistical analyses were conducted using JMP software Student Edition 18.2.2 (SAS Institute, Cary, NC, USA), and a two-sided *p*-value < 0.05 was considered statistically significant. Given the exploratory nature of this study, no adjustment for multiple comparisons was performed, and all findings should be interpreted as hypothesis-generating.

## 3. Results

**Figure 1:**
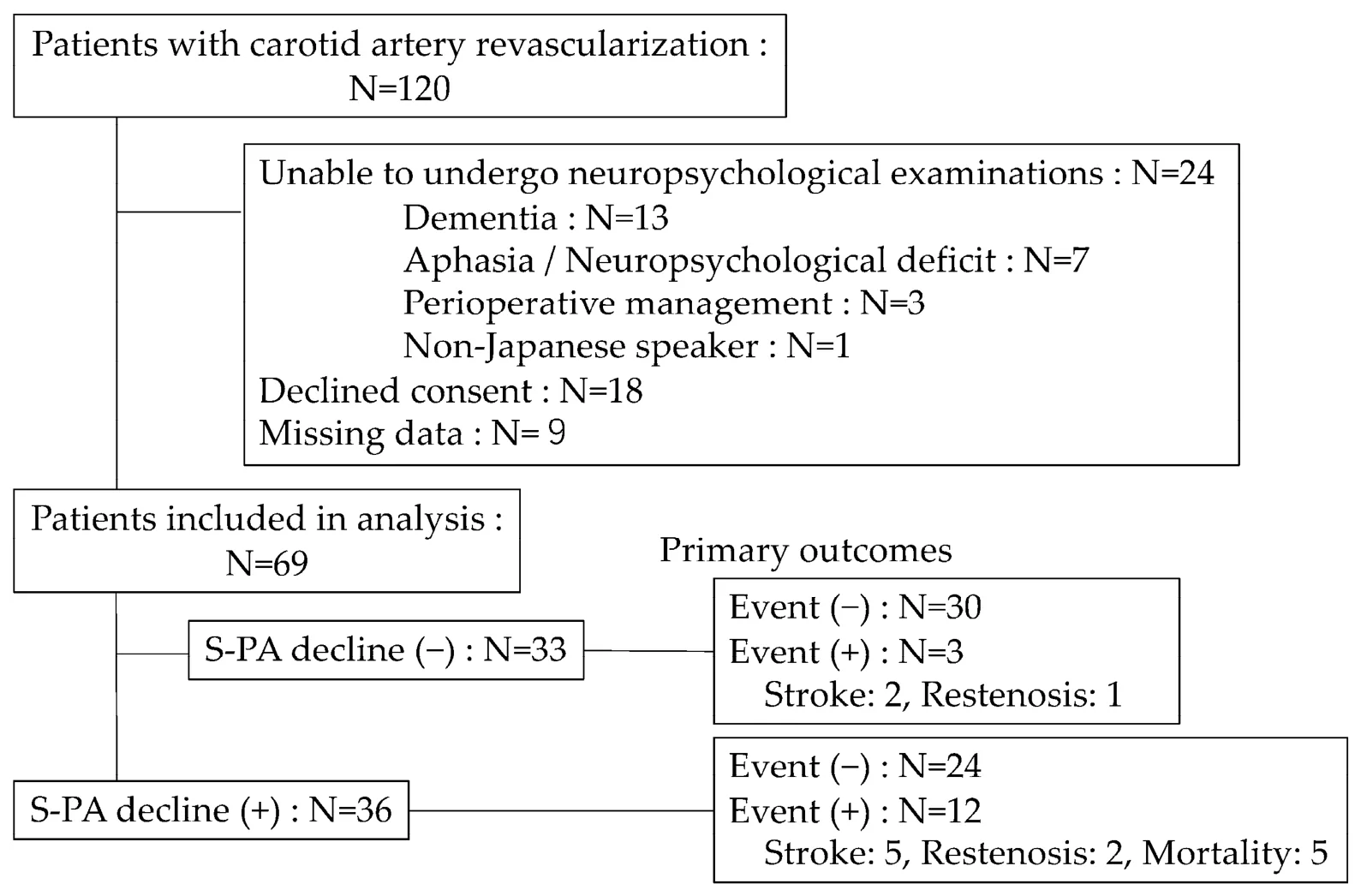
Flowchart of study participants.

Of 120 patients who underwent carotid revascularization during the study period, 69 were included in the final analysis and 51 were excluded. The most common reasons for exclusion were severe dementia (n = 13), aphasia or neuropsychological deficits (n = 7), refusal to provide consent (n = 18), and missing data (n = 9). Among the 69 patients, 15 experienced adverse outcomes during outpatient follow-up, including seven recurrent strokes, three cases of restenosis requiring retreatment, and five deaths (Figure 1).

As shown in Table 1, baseline characteristics such as age (74.5 ± 4.7 vs. 71.9 ± 7.6 years, *p* = 0.457) and sex distribution (male: 100% vs. 92.6%, *p* = 0.569) showed no significant differences between the two groups. None of the evaluated comorbidities showed any significant association with adverse outcomes during outpatient follow-up.

When perioperative cognitive changes in the S-PA domain were examined, 36 patients demonstrated a decline in S-PA, whereas 33 did not. As summarized in Table 2, patients with S-PA decline tended to have lower post-operative mean cerebral blood flow (CBF) compared with those without decline (43.2 ± 6.0 vs. 46.4 ± 6.4 mL/100 g/min, *p* = 0.056). No significant association was observed between age (*p* = 0.154) or sex (*p* > 0.999).

In multivariate analysis, S-PA decline was associated with adverse outcomes in the multivariable Cox model, with a hazard ratio of 4.01 (95% CI: 1.01–15.91, *p* = 0.048). Other factors, including age (HR 1.04, 95% CI: 0.94–1.15, *p* = 0.471) and post-operative mean CBF (HR 1.00, 95% CI: 0.95–1.05, *p* = 0.268), were not identified as significant predictors, as presented in Table 3.

**Figure 2:**
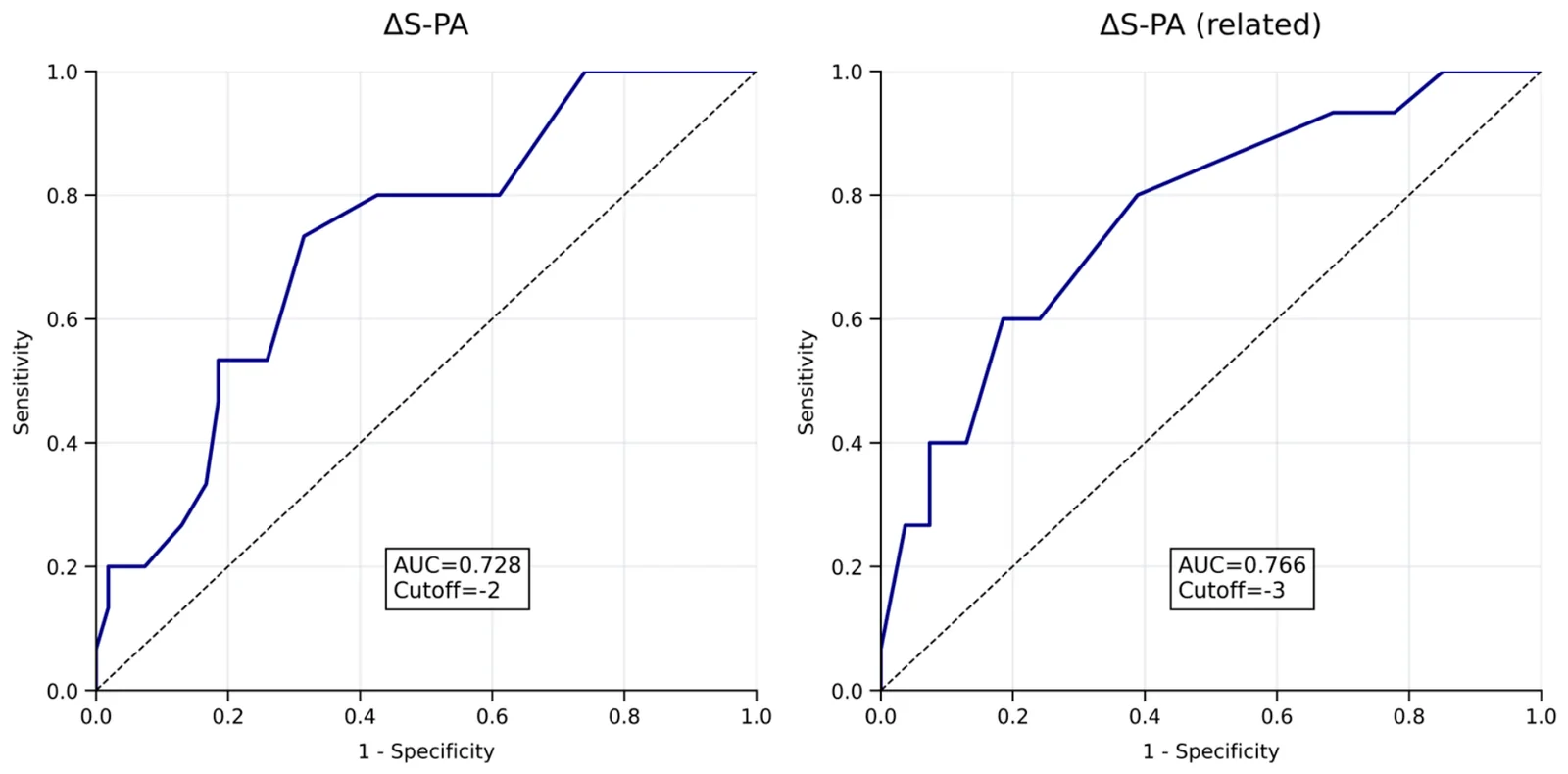
Comparison of receiver operating characteristic (ROC) curves for ΔS-PA and ΔS-PA (related) associated with adverse events.

The preoperative S-PA (related + unrelated) score is 33.8/60, and the postoperative score is 33.0/60. However, the average preoperative S-PA (related) score was 27.0/30, whereas the S-PA (unrelated) score was only 6.8/30, indicating a significant difference in difficulty. Receiver operating characteristic (ROC) analysis was performed to evaluate the predictive ability of ΔS-PA and ΔS-PA (related) for adverse events. The AUC of ΔS-PA was 0.729, indicating moderate discrimination. The optimal cutoff determined by the Youden index was a decline of 2 points (ΔS-PA ≤ −2), providing a sensitivity of 73.3% and a specificity of 68.5%. The median ΔS-PA was −1 point (IQR −3 to +2; range −13 to +14). For ΔS-PA (related), the ROC analysis demonstrated a slightly higher discriminative ability, with an ACU of 0.766. The optimal cutoff determined by the Youden index was a decline of 3 points (ΔS-PA ≤ −3), providing a sensitivity of 60.0% and a specificity of 81.5%. The median ΔS-PA was 0 points (IQR −3 to +1; range −9 to +16). When comparing the two parameters, ΔS-PA (related) showed a higher AUC and specificity, whereas ΔS-PA demonstrated greater sensitivity. These findings suggest that ΔS-PA may be more suitable for screening purposes, while ΔS-PA (related) may be preferable when a higher specificity is desired (Figure 2).

**Figure 3:**
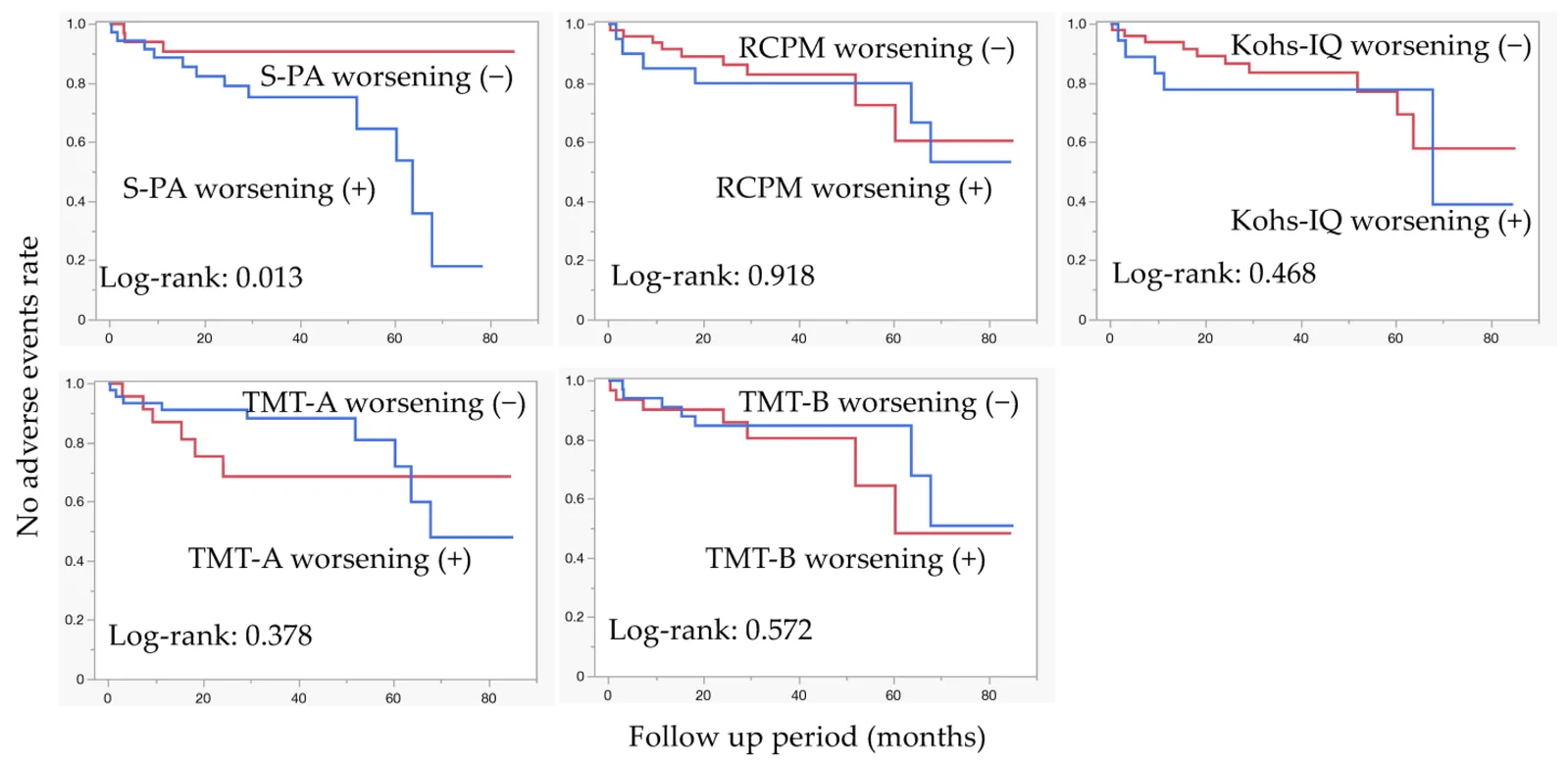
Kaplan–Meier analysis demonstrating adverse event–free survival according to perioperative decline in neuropsychological examinations. Only S-PA decline was significantly associated with adverse outcomes (log-rank *p* = 0.013).

Kaplan–Meier survival analyses further revealed that patients with S-PA decline had significantly lower adverse event-free survival than those without decline (*p* = 0.013, log-rank test). In contrast, no significant differences were observed when survival was analyzed according to the decline in the RCPM (*p* = 0.918), Kohs-IQ (*p* = 0.468), TMT-A (*p* = 0.378), or TMT-B (*p* = 0.572) (Figure 3).

## 4. Discussion

This prospective exploratory study examined the association between perioperative cognitive changes following carotid artery revascularization and subsequent clinical outcomes. We found that patients who experienced a perioperative decline in S-PA tended to have a higher incidence of recurrent stroke, restenosis, and mortality during follow-up. Although any decline was used in the primary exploratory analysis, the ROC analysis suggested that a decline of 2 points may represent a more clinical threshold. These findings have significant implications in clinical practice and may aid in risk stratification and the identification of patients who may require more aggressive secondary prevention strategies.

Previous studies identified PSCI as a significant predictor of recurrent stroke or mortality [11,12]. One meta-analysis of 27 studies comprising 39,412 patients found that PSCI was associated with a statistically significant 59% increased risk of recurrent stroke and a two-fold higher risk of mortality [10]. However, some of the reported discrepancies may be attributed to the study methodology, including population samples, the severity of cognitive impairment, time of the study, timing of neuropsychological assessments, use of non-standardized neuropsychological assessments to determine a cognitive profile, and inconsistent diagnostic classifications of stroke [20]. Our study, which focused on the pathology of carotid artery stenosis that causes atherothrombotic cerebral infarction and chronic hypoperfusion of the brain, showed that carotid revascularization may improve cerebral blood flow without the direct brain manipulation associated with surgery. Therefore, it was possible to evaluate the impact of changes in cerebral blood flow on cognitive function.

Several potential mechanisms may underlie the association between the perioperative decline in S-PA and adverse clinical outcomes. First, the S-PA assesses verbal paired-associate learning, which relies on hippocampal and medial temporal lobe networks known to be particularly vulnerable to chronic cerebral hypoperfusion and microvascular injury [21,22]. Perioperative S-PA decline may thus serve as a sensitive indicator of subclinical brain injury that is not readily detectable on conventional neuroimaging but may predispose patients to subsequent cerebrovascular events. Alternatively, both S-PA decline and recurrent stroke may share common upstream risk factors such as extensive atherosclerotic burden, microembolic load during the procedure, or impaired cerebrovascular reserve. It is important to note that the present study could not establish whether S-PA decline plays a causal role in adverse outcomes or merely serves as a surrogate marker of underlying cerebrovascular vulnerability. Therefore, the observed association should be interpreted as hypothesis generating, and the potential clinical utility of S-PA as a prognostic tool requires validation in prospective studies designed to address this question.

Cognitive outcomes following carotid artery revascularization, including CEA or CAS, have revealed cognitive improvements in patients with severe carotid stenosis, particularly in attention, verbal fluency, and memory within one year of the procedure [4]. In contrast, an increased risk of cognitive impairment after carotid artery revascularization is associated with hyperperfusion, degree of ICA stenosis, diabetes, concomitant intracranial stenosis, and plaque inflammation [23,24]. In the present study, the perioperative S-PA worsening group showed a tendency toward lower mCBF, indicating that hyperperfusion syndrome was unlikely to be affected. Such cognitive changes in carotid artery revascularization are heterogeneous, suggesting that the effect of these interventions depends on appropriate patient selection and individual factors such as age, baseline cognitive function, and vascular comorbidities. Perioperative testing offers the advantage of eliminating the effects of rehabilitation recovery and other diseases associated with the deterioration of higher brain function. However, there are drawbacks such as limited test batteries due to general anesthesia, patient physical strength, and a ceiling effect caused by repeating similar tests within a short period of time. However, the optimal timing and battery of tests for this evaluation remain unclear.

The Japanese Society for Higher Brain Dysfunction developed the S-PA in 2014 to replace the Miyake-Paired Verbal Association Test (University of Tokyo Brain Research Institute Memory Test), which has long been used in Japan. Three standardized sets were available, making it less susceptible to learning effects even when similar tests were administered at different times, thereby enabling a more accurate memory assessment. The S-PA has previously been reported to be highly correlated with the results of the Verbal Memory test and Verbal Paired Association subtest of the Wechsler Memory Scale—Revised (WMS-R) [19]. Furthermore, it is a domain-specific verbal memory test that can be used for comparative studies and implementations. The S-PA is a simple test that can be performed perioperatively and may improve outcomes by initiating rehabilitation, strict management of lifestyle-related diseases, and early regular follow-up. And the result suggest that evaluation is also possible using the more concise S-PA (related) test.

The major limitations of this study were the small sample size (n = 69) and small number of outcome events (n = 15) relative to the number of variables considered. A formal assessment of the proportional hazard’s assumption was not performed. Given the limited sample size and exploratory nature of this study, the Cox model results should be interpreted cautiously. Indeed, the event-per-variable ratio in our multivariable analysis was below commonly accepted standards, resulting in potentially unstable effect estimates, as reflected by the wide confidence intervals. Therefore, our results do not establish S-PA decline as an independent predictor but rather indicate a possible association that warrants further investigation.

Another important limitation relates to the definition and timing of perioperative cognitive assessment. Different postoperative assessment timings between CEA and CAS may have introduced measurement bias. Repeated administration within a short interval may have resulted in a retest effect. Neuropsychological testing within one week of surgery may be influenced by various non-neurological factors such as fatigue, pain, sleep disturbances, and environmental changes. Furthermore, defining S-PA decline as any decrease in the score may overestimate clinically meaningful cognitive deterioration owing to measurement variability and retest effects. A serial neuropsychological follow-up was not performed. Therefore, we could not determine whether the observed S-PA decline represented a permanent deficit or a transient postoperative phenomenon. However, the S-PA provides three standardized versions, partially mitigating this concern. Future studies should therefore incorporate a standardized threshold for clinically meaningful changes and repeat assessments over time.

The definition of study endpoints also merits consideration. In this analysis, we focused on recurrent stroke, restenosis, and all-cause mortality as the primary outcomes as these represent clinically meaningful endpoints with established relevance in carotid revascularization studies. The composite endpoint combined outcomes with different degrees of clinical significance and pathophysiological mechanisms. Asymptomatic restenosis may not have equivalent clinical importance compared with recurrent stroke or mortality. However, restenosis is associated with a high risk of recurrent stroke owing to its heterogeneous clinical implications and frequent asymptomatic detection. In this study, separate multivariable analysis for each endpoint was not performed because of the very small number of events within each category.

Selection bias represents an additional limitation. The treatment choice between CEA and CAS was not randomized and was found to be influenced by plaque characteristics and clinical judgment. Moreover, important variables such as educational attainment, baseline cognitive function, white matter disease burden, medication adherence, perioperative microembolic lesions, and postoperative blood pressure management were not systematically collected. And secondary prevention strategies were not fully accounted for outpatient follow-up. Therefore, the observed association between S-PA decline and adverse outcomes may reflect an underlying cerebrovascular vulnerability, rather than a causal relationship.

Despite these limitations, this study had several strengths. Firstly, the prospective design and standardized perioperative neuropsychological assessment allowed for the systematic evaluation of early cognitive changes, which are commonly overlooked in clinical practice. Patients with severe dementia or neurological deficits were preferentially excluded. Therefore, the analyzed cohort may represent a relatively healthier subgroup, limiting its generalizability. Our findings further indicate that subtle perioperative cognitive decline, particularly in verbal associative memory, may serve as a marker of brain vulnerability. Future multicenter studies with larger sample sizes, standardized cognitive assessment protocols, and adequate statistical power are required to validate these findings and determine whether perioperative cognitive testing can aid in the risk stratification of patients undergoing carotid artery revascularization.

## 5. Conclusions

This exploratory study indicates that a perioperative decline in S-PA following carotid artery revascularization may be associated with adverse outcomes and may reflect underlying cerebrovascular vulnerability. However, owing to methodological limitations, including a small sample size, potential confounding factors, and measurement variability, these findings should be interpreted as hypothesis-generating. Future large-scale prospective studies are needed to clarify the clinical utility of perioperative cognitive assessments.

## Figures and Tables

**Table 1:** Univariate analysis of risk factors associated with adverse outcomes during outpatient follow-up.

		Adverse Outcomes		Univariate Analysis
	Total = 69	Yes = 15	No = 54	*p* Value
Age	72.5 ± 7.1	74.5 ± 4.7	71.9 ± 7.6	0.457
Gender (male)	65 (94.2)	15 (100)	50 (92.6)	0.569 ^†^
Comorbidity				
Hypertension	58 (84.1)	14 (93.3)	44 (81.5)	0.434 ^†^
Hyperlipidemia	52 (75.4)	13 (86.7)	39 (72.2)	0.326 ^†^
Diabetes mellitus	38 (55.1)	9 (60.0)	29 (53.7)	0.665
Coronary disease	24 (34.8)	4 (26.7)	20 (37.9)	0.550 ^†^
Atrial fibrillation	4 (5.8)	1 (6.7)	3 (5.6)	>0.999 ^†^
Current smoker	53 (77.9)	10 (71.4)	43 (79.6)	0.491 ^†^
Symptomatic	40 (57.9)	8 (53.3)	32 (59.3)	0.681
Left-side lesion	30 (43.5)	5 (33.3)	25 (46.3)	0.126
NASCET Mean ± SD (%)	76.8 ± 13.9	80.0 ± 10.5	76.0 ± 14.7	0.374
Treatment: CEA	52 (75.4)	10 (66.7)	42 (77.8)	0.377
Post-op hyperperfusion	1 (1.5)	0	1 (1.85)	>0.999 ^†^
Post-op MRI DWI HIA	27 (39.1)	7 (46.7)	20 (37.0)	0.499
Decline of cognitive function				
RCPM	20 (28.9)	6 (40.0)	14 (25.9)	0.288
Kohs-IQ	18 (26.5)	5 (33.3)	13 (24.5)	0.495
TMT-A	46 (66.7)	9 (60.0)	37 (68.5)	0.549
TMT-B	35 (53.0)	7 (46.7)	28 (53.9)	0.798
S-PA	36 (52.2)	12 (80.0)	24 (44.4)	0.020 ^†^
Follow-up period (Month)	35.9 ± 21.6	24.5 ± 24.3	39.1 ± 20.8	0.0092

^†^: Fisher’s exact test.

**Table 2:** Univariate analysis of risk factors for total S-PA worsening.

	Deterioration of Total S-PA		Univariate Analysis
	Yes = 36	No = 33	*p* Value
Age	73.8 ± 6.6	71.1 ± 7.5	0.154
Gender (male)	34 (94.4)	31 (93.9)	>0.999 ^†^
Post-op mCBF (mL/100 g/min)	43.2 ± 6.0	46.4 ± 6.4	0.056
Adverse Events	12 (33.3)	3 (9.1)	0.020
Mortality	5 (13.9)	0	0.055

^†^: Fisher’s exact test.

**Table 3:** Hazard ratio of adverse outcomes during outpatient follow-up.

Variable	Hazard Ratio (95% CI)	*p* Value
Age	1.04 (0.94–1.15) *	0.471
Postoperative mCBF	1.00 (0.95–1.05) *	0.268
Symptomatic	2.4 (0.67–8.74)	0.175
S-PA worsening	4.01 (1.01–15.91)	0.048

*: unit hazard ratio.

## Data Availability

The datasets generated during and/or analyzed during the current study are available from the corresponding author upon reasonable request due to patient privacy and confidentiality constraints.

## Abbreviations

The following abbreviations are used in this manuscript:

CAS	carotid artery stenting
CBF	cerebral blood flow
CEA	carotid endarterectomy
DWI	diffusion-weighted images
HIA	high-intensity areas
KBCT	Kohs Block Design Test
MRA	magnetic resonance angiography
MRI	magnetic resonance imaging
PSCI	post-stroke cognitive impairment
RCPM	Raven’s Colored Progressive Matrices
S-PA	Standard Verbal Paired Associate Learning Test
SPECT	single-photon emission computed tomography
TMT-A and B	Trail Making Test—A and B
WMS-R	Wechsler Memory Scale—Revised
